# Improving Clinical Manufacturing of IL-15 Activated Cytokine-Induced Killer (CIK) Cells

**DOI:** 10.3389/fimmu.2019.01218

**Published:** 2019-05-31

**Authors:** Melanie Bremm, Lisa-Marie Pfeffermann, Claudia Cappel, Verena Katzki, Stephanie Erben, Sibille Betz, Andrea Quaiser, Michael Merker, Halvard Bonig, Michael Schmidt, Thomas Klingebiel, Peter Bader, Sabine Huenecke, Eva Rettinger

**Affiliations:** ^1^Clinic for Pediatric and Adolescent Medicine, University Hospital, Frankfurt, Germany; ^2^Department of Cell Therapy, Fraunhofer Institute for Cell Therapy and Immunology IZI, Leipzig, Germany; ^3^Division for Translational Development of Cellular Therapeutics, Institute for Transfusion Medicine and Immunohematology, Goethe-University Frankfurt, Frankfurt, Germany

**Keywords:** CIK cells, immunotherapy, allogeneic stem cell transplantation, cryopreservation, AB-serum, fresh frozen plasma, platelet lysate

## Abstract

Cytokine-induced killer (CIK) cells are an immunotherapeutic approach to combat relapse following allogeneic hematopoietic stem cell transplantation (HSCT) in acute leukemia or myelodysplastic syndrome (MDS) patients. Prompt and sequential administration of escalating cell doses improves the efficacy of CIK cell therapy without exacerbating graft vs. host disease (GVHD). This study addresses manufacturing-related issues and aimed to develop a time-, personal- and cost-saving good manufacturing process (GMP)-compliant protocol for the generation of ready-for-use therapeutic CIK cell doses starting from one unstimulated donor-derived peripheral blood (PB) or leukocytapheresis (LP) products. Culture medium with or without the addition of either AB serum, fresh frozen plasma (FFP) or platelet lysate (PL) was used for culture. Fresh and cryopreserved CIK cells were compared regarding expansion rate, viability, phenotype, and ability to inhibit leukemia growth. Cell numbers increased by a median factor of 10-fold in the presence of FFP, PL, or AB serum, whereas cultivation in FFP/PL-free or AB serum-free medium failed to promote adequate CIK cell proliferation (*p* < 0.01) needed to provide clinical doses of 1 × 10^6^ T cells/kG, 5 × 10^6^ T cells/kG, 1 × 10^7^ T cells/kG, and 1 × 10^8^ T cells/kG recipient body weight. CIK cells consisting of T cells, T- natural killer (T-NK) cells and a minor fraction of NK cells were not significantly modified by different medium supplements. Moreover, neither cytotoxic potential against leukemic THP-1 cells nor cell activation shown by CD25 expression were significantly influenced. Moreover, overnight and long-term cryopreservation had no significant effect on the composition of CIK cells, their phenotype or cytotoxic potential. A viability of almost 93% (range: 89–96) and 89.3% (range: 84–94) was obtained after freeze-thawing procedure and long-term storage, respectively, whereas viability was 96% (range: 90-97) in fresh CIK cells. Altogether, GMP-complaint CIK cell generation from an unstimulated donor-derived PB or LP products was feasible. Introducing FFP, which is easily accessible, into CIK cell cultures was time- and cost-saving without loss of viability and potency in a 10-12 day batch culture. The feasibility of cryopreservation enabled storage and delivery of sequential highly effective ready-for-use CIK cell doses and therefore reduced the number of manufacturing cycles.

## Introduction

Adoptive immunotherapy with donor-derived cytokine-induced-killer (CIK) cells is a promising approach to counteract impending relapse after allogeneic hematopoietic stem cell transplantation (HSCT) in patients with hematological malignancies. CIK cells have shown potent anti-leukemic cytotoxicity *in vitro* and *in vivo* animal models while demonstrating only low alloreactive potential (1–8). By virtue of their heterogeneous cell composition, including a majority of CD3^+^CD56^−^ T cells and CD3^+^CD56^+^ T- natural killer (T-NK) cells and a minor contribution of CD3^−^CD56^+^ NK cells, CIK cells can mediate both T cell receptor dependent and non-major histocompatibility complex (MHC)-restricted cytotoxicity (7, 9, 10). Their killing activity is mediated by different mechanisms involving several receptors including NKG2D, TRAIL, FasL, DNAM-1, NKp30, LFA-1, and perforin/granzyme secretion (5, 11–13).

Adoptive cell immunotherapy might be used with the aim to further improve survival in patients suffering from impending relapse indicated by upcoming minimal residual disease (MRD) or mixed chimerism in the post-transplant period. CIK cells can be prepared from leukocytapheresis material, peripheral blood, bone marrow, or even cord blood mononuclear cells in the presence of interferon IFN-γ, anti-CD3 antibody and interleukin (IL)-2. The general culture protocol requires 3 weeks. Considering the urgent need for treatment options of these patients, we recently focused on generating a highly effective CIK cell product within just 10–12 days (14). By adding IL-15 from day 4 of culture, cells with CIK cell phenotype were expanded within 10–12 days. *In vitro* cytotoxicity of IL-15 activated CIK cells was significantly enhanced compared to IL-2 activated controls and *in vivo* anti-leukemic efficacy extensively tested in several established mouse models of human leukemia xenografts, showed homing of IL-15 activated CIK cells to leukemia sites, leukemia control, and when repeatedly given complete disease clearance indicated by improved survival (15). Furthermore, CIK cells were effective in killing solid tumors, including chemoresistant cancer stem cells (13, 16, 17).

**Figure 1:**
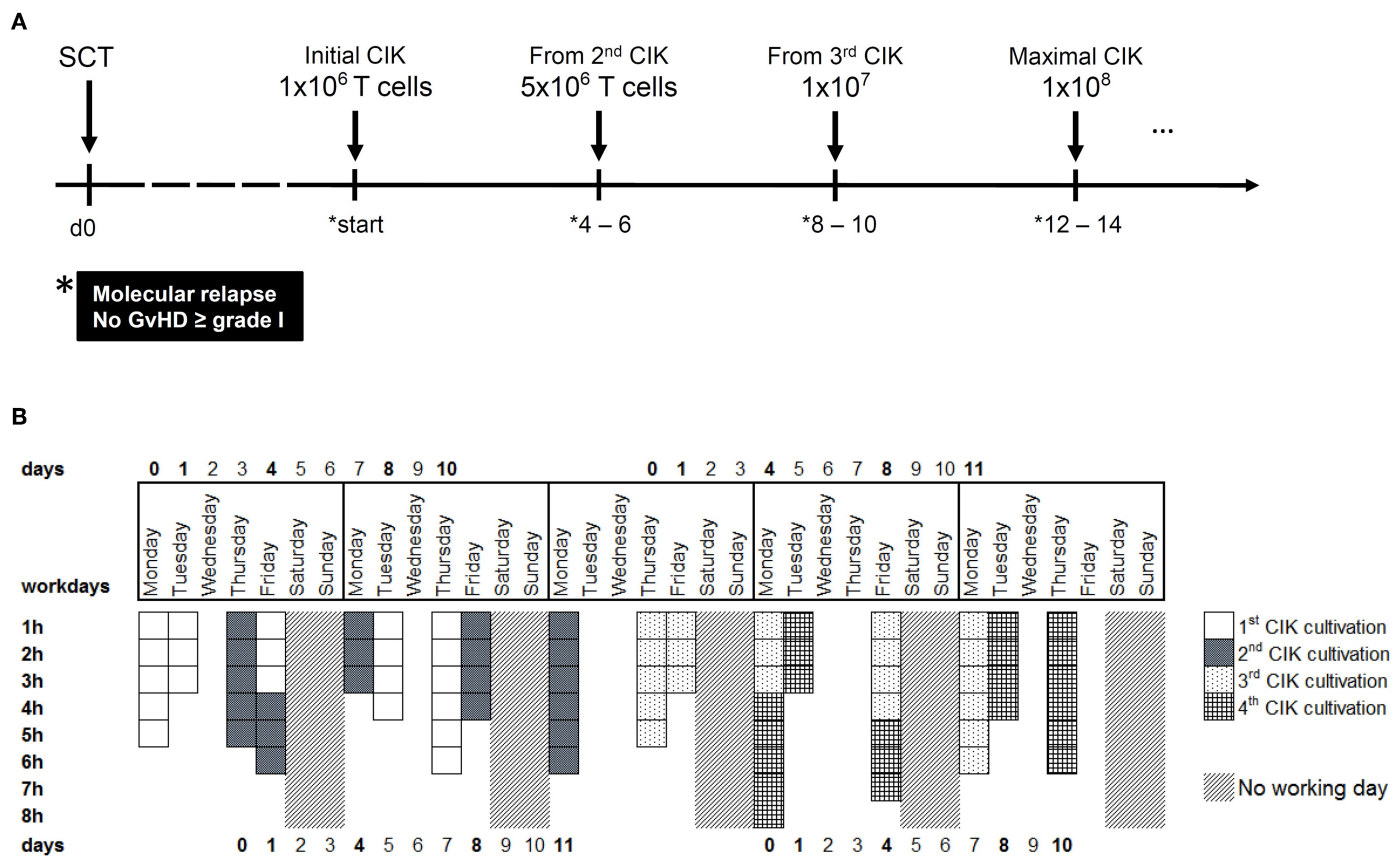
CIK cell generation within the clinical phase I/II study. **(A)** CIK cells are administered to patients with molecular relapse defined as mixed chimerism ≥1% of recipient (autologous) signals; detectable MRD ≥10^−6^ or BCR-ABL/ABL ≥10^−4^. According to ATMP § 4b license, repeated CIK cell applications with escalating doses may be provided to patients suffering from prolonged molecular relapse in intervals of 4–6 weeks under the condition that no GVHD ≥ grade I occurred. **(B)** Within a time-frame of 5 weeks, four different batches of CIK cells can be manufactured. The working days and the estimated time for tasks like initiation, cytokine stimulation or harvest are shown.

Following good manufacturing practice (GMP)-compliant procedures, CIK cells were than time and time again expanded from 50 to 200 mL peripheral blood products of original stem cell donors and given as a single dose with a maximum of 1 × 10^8^ T cells/kG body weight to respective patients. Of note, each CIK cell manufacturing is complex and labor, personal, and cost intensive. To prepare for a growing number of patients in need several infusions for immediate use, a robust GMP-compliant process yielding several clinical doses of 1 × 10^6^ T cells/kG, 5 × 10^6^ T cells/kG, 1 × 10^7^ T cells/kG, and 1 × 10^8^ T cells/kG recipient body weight had to be established (Figure 1A). In this context, the manufacturing process of CIK cells for which the authors are holding the license and marketing authorization (Advanced therapy medicinal product (ATMP) § 4b Abs. 3 AMG, license number: PEI.A.11630.01.1) was amended. The concept of cellular therapy product manufacturing presented here encompasses the expansion of individual patient doses from donor-derived peripheral blood (PB) or leukocytapheresis (LP) products. Additional issues addressed by this study were the generation of CIK cells from peripheral blood stem cells (PBSC), cell culture media and supplements used for expansion. Commercially available serum-free media usually contain high amounts of human serum albumin, which should make serum supplementation redundant to comply with GMP standards. However, expansion of CIK cells so far was reported only in the presence of serum or plasma (1, 9, 14). But, within clinical routine we were often confronted with bottlenecks of AB serum so that manufacturing slots had to be postponed or even canceled, which prompted us to search for different media supplements. Furthermore, since CIK cells might be affected by cryopreservation and thawing, with loss of cell numbers, viability and function, cell recovery after cryopreservation was also a scope of this manuscript.

To enable clinical application of sequential CIK cell therapy given in escalating doses to patients with relapsing hematological malignancies after allogeneic HSCT, the present study aimed to establish GMP-compliant procedures for robust generation of therapeutic CIK cell doses. CIK cells were expanded form PB, LP, or PBSC products. Since the clinical application of CIK cells will most probably include media supplements for *in vitro* expansion, different additives were analyzed to retain cell proliferation, activation and potency of CIK cells. Given that CIK cells had to be stored and shipped, it was of importance to determine cell profiles after freeze-thawing experiments.

## Materials and Methods

### CIK Cell Generation

**Figure 2:**
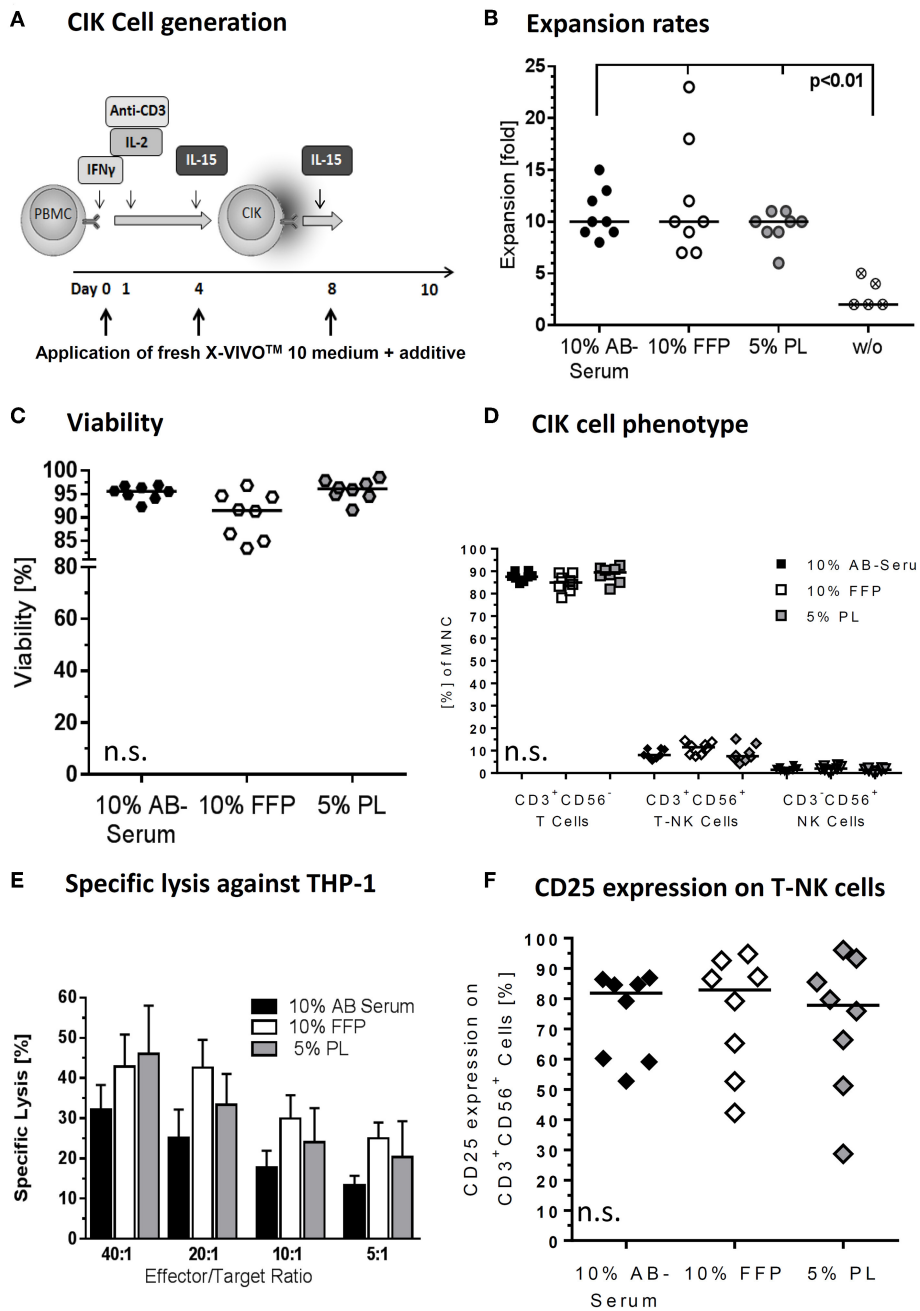
Medium supplements for CIK cell cultivation. **(A)** CIK cells are supplemented with fresh media including different additives on days 0, 4, and 8 of culture. We tested different additives including fresh frozen plasma (FFP) and platelet lysate (PL) in different concentrations to substitute AB serum in the cultivation process. **(B)** With median 10-fold expansion rates, CIK cell cultivation with 10% AB serum, 10% FFP and 5% PL was similar. CIK cell expansion without additive was significantly lower compared to cultivation with medium supplement. **(C)** With 91.5% viability was minimal lower when CIK cells were cultured with FFP compared to AB serum (95.6%) or PL (96.1%), but not statistically relevant. **(D)** The composition of CIK cells consisting of T-, T-NK-, and NK-cells was not significantly influenced by the different cultivation variants. **(E)** CIK cell cytotoxicity was tested against the leukemic cell line THP-1. In the effector/target ratio of 40:1 mean specific lysis was with 46.0% highest for PL-CIK cells, closely followed by FFP-CIK cells with 42.9%. In the effector/target ratios 20:1, 10:1, and 5:1, CIK cells generated with FFP as additive showed the highest cytotoxicity. **(F)** CD25 expression on T-NK cells was highest on CIK cells cultivated with FFP. Comparable results were obtained for CIK cells cultured with AB serum. CIK cells cultivated with PL as medium additive showed a slightly lower CD25 expression and a wider range of expression. *n* = 8 experiments, except cultivation without medium supplement (*n* = 5) and cytotoxicity analysis (*n* = 3).

This study was carried out in accordance with the recommendations of the medical ethics committee, with written informed consent from all subjects. All subjects gave written informed consent in accordance with the Declaration of Helsinki. The protocol was approved by the medical ethics committee of the University Hospital Frankfurt (Ref. No. 281/14). For research, CIK cells were generated from either PB, unstimulated LP (fresh and cryopreserved) or cryopreserved PBSC of healthy donors of whom we obtained written informed consent according to the guidelines of the. In the case of PB, PBMCs were obtained by ficoll density centrifugation. The cells were adjusted to 3 × 10^6^ cells/ml and cultured in X-VIVO 10 media (Lonza, Verviers, Belgium). Afterwards, cells were supplemented with one of the following additives, respectively: 10% heat-inactivated AB serum (German Red Cross Blood Donor Service, Tübingen, Germany), 10% heat-inactivated fresh frozen plasma (FFP) (German Red Cross Blood Donor Service, Frankfurt, Germany), 5% PL (German Red Cross Blood Donor Service, Frankfurt, Germany), 10% XerumFree^TM^ (serum free herbal additive, TNC Bio BV, Eindhoven, Netherlands), 10% XerumFree^TM^ + 0,3% Human Serum Albumin (HSA, albunorm 20%, Octapharma, Lachen, Switzerland). At day 0 of CIK generation, 1,000 U/ml IFN-γ (Imukin®, Boeringer Ingelheim Pharma, Germany) were added. On day one 100 ng/ml anti-CD3 mAB (OKT-3, MACS GMP CD3 pure, Miltenyi Biotec, Bergisch-Gladbach, Germany) and 500 U/ml IL-2 (Proleukin®, Novartis Pharma, Nuremberg, Germany) were added. CIK cell density was adjusted to 1 × 10^6^/ml on days 4 and 8 and the cells were re-stimulated with 50 ng/ml IL-15 (PeproTech, Rocky Hill, USA). CIK cells were harvested following 10 days of culture (Figure 2A).

Regarding clinical application, sequential CIK cell applications with escalating cell doses were manufactured according to ATMP § 4b (Figure 1A). CIK cells were generated either from PB or LP using VueLife® FEP Bag 750-C1 (Cellgenix) and clamping up at least half of the bag at day 0 until large cell colonies were visible and additional culture medium was needed.

### Cryopreservation of CIK Cells

To evaluate cell viability and cytotoxic activity, part of the harvested CIK cells was cryopreserved overnight. Furthermore, long-term storage of at least 1 month (median storage time: xx months; range: xx–xx months) was investigated by thawing n = x CIK samples which have not been infused during patients' lifetime. For cryopreservation, freshly harvested CIK cells were diluted with HSA (Octapharma, Lachen, Switzerland) including Dimethyl Sulfoxide (DMSO, CryoSure 50, WAK-Chemie, Steinbach/Ts, Germany) in a concentration of 7.5% in the final product. The whole process was performed on cooling elements to avoid cell death induced by DMSO. Afterwards, the CIK cell dilution was cryopreserved in freezing bags (CryoMACS Freezing Bag, Miltenyi Biotec, Bergisch-Gladbach, Germany) using controlled-rate BIOFREEZE® BV45 freezing machine (Consarctic, Schoellkrippen, Germany). The cryopreserved CIK cells were stored overnight in liquid nitrogen. On the next day, cells were thawed using Barkey plasmatherm thawing device (Barkey, Leopoldshoehe, Germany). Afterwards, cells were washed with NaCl (NaCl 0.9%, Braun, Melsungen) + 0.5% HSA and transferred into culture flasks filled with preheated X-VIVO 10 media including 5% HSA.

### Europium Release Cytotoxicity Assay

The killing activity of CIK cells was analyzed in a non-radioactive europium release assay against the leukemic cell lines THP-1 and K562 as described previously (18, 19). In short, target and CIK cells were co-cultured in triplicates at effector to target (E:T) ratios of 40:1, 20:1, 10:1, and 5:1. After 3 h of co-incubation at 37°C supernatants were collected and incubated with Europium solution (PerkinElmer, Boston, USA). Maximum release (positive control) was obtained by target cell incubation with Triton™ X-100 solution (Sigma-Aldrich Chemie, Steinheim, Germany), whereas target cells without effector cells were used as negative control. The fluorescence signal, correlating with killing activity, was measured by a multilabel plate reader (VICTOR^3™^ 1420 multilabel counter, PerkinElmer, Boston, USA).

### Trypan Blue Viability Analysis

Within the clinical grade generation, CIK cell viability after cryopreservation and thawing was tested via trypan blue microscopic analysis. In short, 20 μl CIK cells and 20 μl of trypan blue solution (Sigma-Aldrich Trypan Blue Stain, 0.4%, Merck, Darmstadt, Germany) was used as a cell stain to assess cell viability using the dye exclusion test.

### Flow-Cytometric Analysis

Flow-cytometric analysis of CIK cells was performed on a Navios™ 10-color flow-cytometer via single platform analysis applying Flow-Count fluorospheres (Beckman Coulter, Brea, USA) on day 0 and after harvesting as described previously (18, 19). Leukocyte counts on day 4 and 8 of culturing were measured on a DxH hematology analyzer (Beckman Coulter, Krefeld, Germany). For flow-cytometry monoclonal antibodies conjugated with fluorescein-isothiocyanate (FITC), phycoerythrin (PE), phycoerythrin-Texas Red® (ECD), phycoerythrin-cyanine-5.5 (PC-5.5), phycoerythrin-cyanine-7 (PC-7), allophycocyanin (APC), APC-Alexa Fluor 700 (APC-A700), APC-Alexa Fluor 750 (APC-A750), Pacific Blue™ (PB), Krome Orange (KO) were used against following antigens (clones): FITC: TCRγδ (IMMU510), CD62L (DREG56); PE: TCRαβ (BW242/412)^1^, CD314/NKG2D (ON72)/(149810)^4^; ECD: CD19 (J3-119), CD45RO (UCHL1); PC-5.5: CD45 (J.33); PC-7: CD56 (N901/NKH-1); APC: CD3 (UCHT1); APC-A700: CD25 (B1.49.9); APC-A750: CD16 (3GB), CD4 (13B8.2); PB: CD14 (RMO52), CD45RA (2H4); KO: CD45 (J.33), CD8 (B9.11) (all mouse IgG1, other than ^#^IgG2a, ^*^IgG2b, all antibodies Beckman Coulter, except ^1^Miltenyi Biotec, ^2^BD Biosciences; ^3^Biolegend, ^4^R&D Systems). CIK cell viability was determined via 7AAD negativity. Data were analyzed using Navios software (Vs. 1.2, Beckman Coulter, Krefeld, Germany).

### Cytokine/Chemokine Analysis

Supernatants of expanded CIK cells after 4, 8, and 10–12 days of cultivation were collected and assayed using BioLegend LEGENDplex^TM^ (BioLegend, San Diego, USA). Data acquisition was performed on a Navios Flow Cytometer and analyzed with the LEGENDplex^TM^ Data Analysis Software (BioLegend, San Diego, USA). The cell density was adjusted to 1 × 10^6^/ml. The human inflammation CD8/NK panel was designed for quantification of the cytokines/chemokines IL-2, IL-4, IL-6, IL-17A, sFas, sFasL, Granzyme A, Granzyme B, Perforin, and Granulysin. The Anti-Virus Response panel was used to detect the cytokines/chemokines IL-1β, TNF-α, IP-10, IL-29, IL-8, GM-CSF, IL-10, IFN-γ, and IL-28A/B. The minimum detectable concentration for the cytokines ranged from 0.6 to 2.1 pg/ml.

### Statistical Analysis

Statistical analysis was performed using GraphPad Prism 6 for Windows (GraphPad Software, San Diego, USA). Data were compared by an unpaired *T*-Test and differences were considered as significant for *p* < 0.05, *p* < 0.01, and *p* < 0.001.

## Results

### GMP-Compliant CIK Cell Expansion

Regarding clinical application, sequential CIK cell applications with escalating cell doses were manufactured and released according ATMP § 4b Abs. 3 AMG, license number: PEI.A.11630.01.1 (Figure 1A). All CIK cell doses should fulfill pre-defined specification in terms of cell viability (specification >70%), cell expansion of CD3^+^CD56^+^ T-NK cells expressing the activation antigen CD25 (specification >10-fold) and T cells amount (specification < 1 × 10^8^/kg) post-preparation. The generated cellular products should further be negative for culturable microbes and free of mycoplasma and endotoxin. The cytokine profile on days 4, 8, and 10–12 during culture is summarized in Supplementary Figure 1.

Altogether, within a period of 5 weeks, we are able to produce CIK cells for four different patients, resulting in a total of 34 CIK cell products per year. The expenditure of time per product is displayed in Figure 1B. However, generation of CIK cells was limited by both clean-room capacities and experienced manufacturing/quality control personnel, respectively.

### Serum-Free Cell Culture Media and Plasma/Serum Substitutes and CIK Cell Expansion and Viability

For clinical application, the CIK cell expansion was adapted to GMP-grade. Nevertheless, CIK cells activated in X-Vivo 10 was supposed to require human plasma/serum supplementation for efficient cell expansion. Therefore, commercially available GMP-grade cell culture media X-Vivo 10 was compared with regard to its ability to promote growth of CIK cells as a plasma/serum-free and plasma/serum-supplemented formulation, also considering bottlenecks in AB serum (Figure 2A). A difference in cell proliferation was not detected. The presence of AB serum, FFP and PL seemed to improve cell growth by 10-fold. The most pronounced effect was observed at days 10–12 with fold expansion of 10 (range: 8–15), 10 (range: 7–23), and 10 (range: 6–11) for X-Vivo 10 containing human AB serum, FFP and PL, respectively. Cell cultivation without supplements resulted in significantly lower CIK cell expansion (*p* < 0.01) (Figure 2B). Similarly low rates were reached applying XerumFree^TM^ + Human Serum Albumin as medium additives (data not shown). Therefore, plasma/serum-free culture might not be efficient in a clinical setting providing significantly lower cell doubling when compared to plasma/serum-supplemented culture.

Since the growth and viability of CIK cells is cytokine-dependent, we aimed to optimize the cytokine cocktail for *ex vivo* cell expansion. The applied cytokine cocktail enabled maintenance of cell viabilities of 95.6% (range: 92.3–96.9%), 91.5% (range: 83.4–96.8%), and 96.1% (range: 91.6–98.6%) for AB serum, FFP and PL supplements during the whole expansion course (Figure 2C).

### Plasma/Serum Substitutes and CIK Cell Phenotype

At the end of the cultivation procedure, the quality of the cells was additionally evaluated and compared for cell compositions in different plasma/serum-supplemented medium. Regarding the composition of CIK cells no significant differences were determined while culturing with different additives. CIK cells consisted in median of 87.6% (range: 84.6–90.3%), 84.9% (range: 78.2–89.3%), 89.4% (range: 82.0–92.6%) T cells, 8.0% (range: 5.7–11.1%), 11.5% (range: 7.5–14.4%), and 7.5% (range: 4.2–15.2%) T-NK cells and 1.5% (range: 0.6–3.4%), 1.9% (range: 0.6–3.7%), and 1.5% (range: 0.6–2.4%) NK cells for AB serum, FFP and PL, respectively (Figure 2D).

### Plasma/Serum Substitutes and Cytotoxicity of CIK Cells

Simultaneously performed functionality assays showed that different medium supplements did not alter the specific cytotoxicity against leukemia target cell line THP-1. All three medium supplements tested resulted in specific cytotoxicity of CIK cells against THP-1 target cells. Mean cytotoxicity of CIK cells against THP-1 was 32.2% (SD: 10.6), 42.9% (SD: 13.8), and 46.0% (SD: 20.8) at an effector to target cell ratio 40:1 for CIK cells cultured with AB serum, FFP and PL, respectively (Figure 2E). Considering effector to target ratios of 20:1, 10:1 and 5:1, CIK cells cultivated in the presence of FFP showed the highest cytotoxicity against THP-1 cells; however no significant differences were determined. Moreover, there was no influence of medium supplements in terms of activation antigen expression CD25 on CD3^+^CD56^+^ T-NK cells. The immunophenotypic characterization of T-NK cells revealed 81.9% (range: 52.7–86.9%) and 82.9% (range: 42.2–94.8%) activation antigen CD25 expression after activation with AB serum and FFP compared to 77.8% (range: 28.7–96.1%) CD25 expression on T-NK cells cultivated with PL (Figure 2F).

The above promising data on expansion, phenotype and function in media supplemented with FFP and PL encouraged us to consider both as alternatives to AB serum supplementation. Although quality results for CIK cells generated with FFP and PL were similar, with regard to the easier accessibility of FFP we decided to approve FFP as supplement for CIK cell expansion. The ATMP § 4b Abs. 3 AMG, license number: PEI.A.11630.01.1 was amended accordingly.

### Cryopreservation of CIK Cells With Regard to Viability

**Figure 3:**
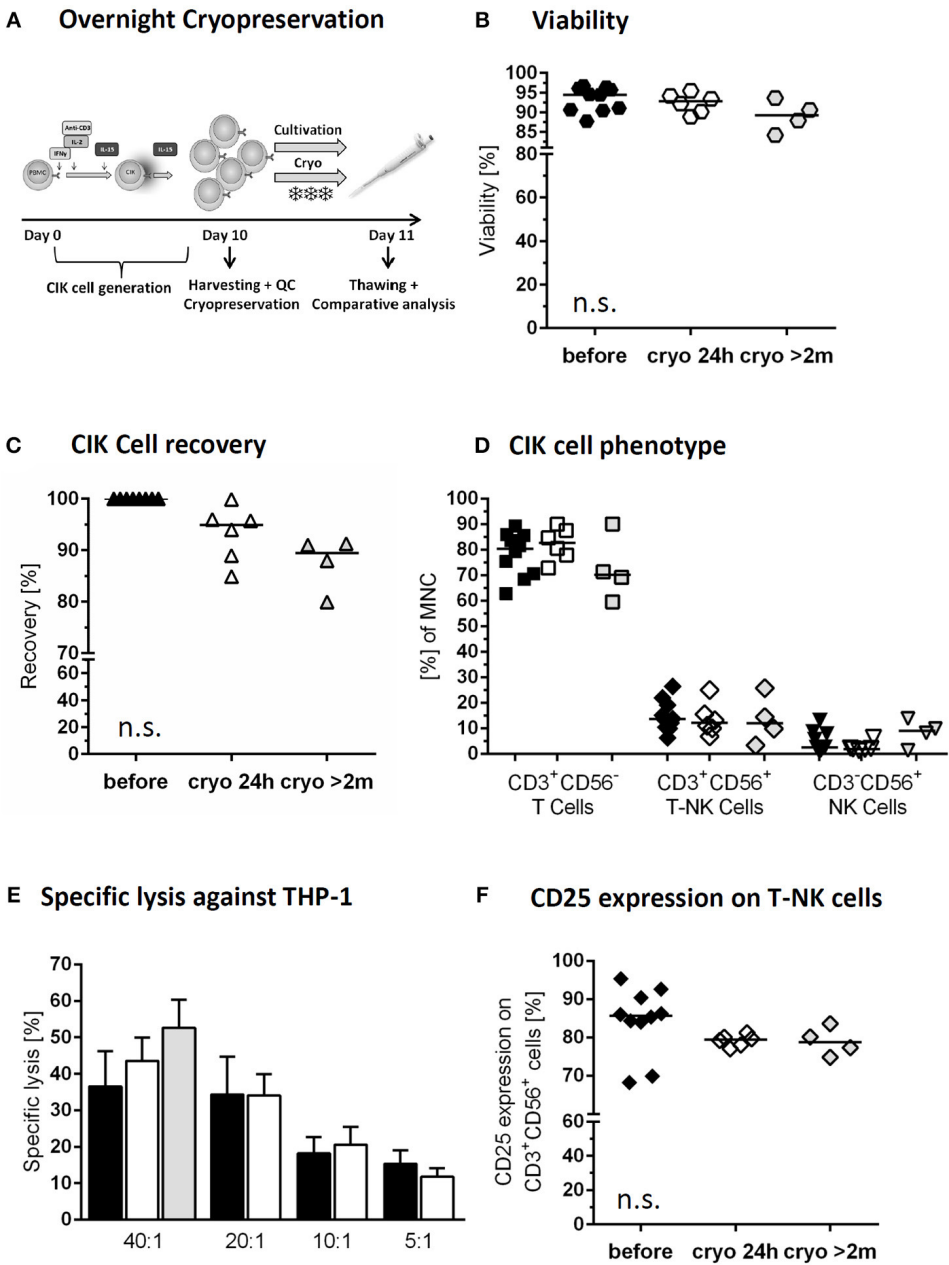
Cryopreservation of CIK cells. Comparison of freshly harvested CIK cells (black) and CIK cells following overnight cryopreservation of the same donor (white). In addition long time storage of at least 2months (median 2 years) was investigated (gray; differing donors) **(A)** On day 10, one part of the freshly harvested CIK cells was cryopreserved overnight, whereas a second part was further cultivated overnight. **(B)** Viability was slightly, but not significantly, reduced due to overnight and long-term cryopreservation. **(C)** CIK cell recovery was 94.9% after thawing following overnight cryopreservation an 89.5% after long-term storage. **(D)** The composition of CIK cells consisting of T-, T-NK- and NK-cells was not significantly influenced by overnight and long-term cryopreservation. **(E)** Cytotoxicity against THP-1 of CIK cells before and after cryopreservation was comparable in the tested effector/target ratios 40:1, 20:1, 10:1, and 5:1 for overnight (*n* = 6) and long-term cryopreservation (*n* = 3). **(F)** Independent of storage time CD25 expression on CD3^+^CD56^+^ cells minimally decreased following cryopreservation, but not statistically relevant. *n* = 6 experiments.

The investigational medicinal product (IMP) with cryopreservation of predefined CIK cell doses might be a suitable strategy to minimize manufacturing time and costs for each patient (Figure 3A). To analyze the function of a patients' cryopreserved CIK cell doses, we tested viability of CIK cells stored under above mentioned overnight cryo- and thawing conditions. Our data demonstrated high stability and viability of the cryopreserved CIK cells with a viability of 95.9% (range: 90.4–96.7%) before and 92.8% (range: 88.9–95.5%) after cryopreservation. Furthermore, we analyzed long-term storage with a median storage time of 2 years (range: 0.2–2.5 years). Also following long-term storage a slightly lower, but satisfying viability of 89.3% (range: 84.2–93.7%) could be shown (Figure 3B).

In total 42 thawing controls were analyzed via trypan blue dye exclusion test since 2016. This analysis is an integral part of the pre-release testing. In all cases the specification of >70% viability was met. On the basis of these results, cryopreservation of CIK cells was granted approved by the National Authority (Paul-Ehrlich-Institute).

### Cryopreservation of CIK Cells and Phenotype

The CIK cell product should preserve its phenotypic features. Indeed, cryopreservation had no significant effect on CIK cell subpopulations. Median recovery of the bulk CIK cell population after overnight storage was 94.9% (range: 84.9–99.9%) and 89.5% (range: 79.99–91.3%) following long-term cryopreservation (Figure 3C). CIK cells in median before cryopreservation vs. overnight and long-term storage consisted of 80.4% (range: 62.8–89.3) vs. 82.7% (range: 72.8–90.1%), and 70.2% (range: 59.6–90.0%) T cells, 13.7% (range: 6.3–26.4%) vs. 12.1% (range: 6.9–25.1%) and 12.1% (range: 3.3–25.7%) T-NK cells and 2.5% (range: 0.9–13.1%) vs. 1.9% (range: 1.2–6.3%) and 9.0% (range: 1.4–13.9%) NK cells, respectively (Figure 3D). The remarkably high content of NK cells within the CIK cells selected for long-term cryopreservation may be explained by also exceptionally high amounts of NK cell within the primary material.

### Cryopreservation of CIK Cells With Regard to Cytotoxicity

Cytotoxicity was not altered by cryopreservation as the killing activity of CIK cells against THP-1 cell line was indistinguishable before and after the process at all tested effector to target ratios. The cytotoxic potential of cryopreserved CIK cells following a median storage time of 2 years was tested against THP-1 in an effector to target ratio of 40:1. The results were slightly, but not significantly higher than those gained for freshly generated CIK cells and those following 24 h cryopreservation. In contrast to freshly generated CIK cells and overnight storage, for long-term cryopreservation CIK cells were gained from different donors (Figure 3E). Following overnight cryopreservation also cytotoxicity against K562 cells war tested with comparable results (data not shown). Activation antigen CD25 expression on CD3^+^CD56^+^ cells was retained, showing in median 85.7% (range: 68.2–95.3%) before, 79.4% (range: 77.0–81.2%) after 24 h cryopreservation and 78.7% (range: 74.8–83.6%) following long-term storage. The increased expression of at least 10-fold from the beginning of CIK cell culture until harvest was retained also over cryopreservation in all cases (Figure 3F).

### Primary Material for CIK Cell Generation

**Figure 4:**
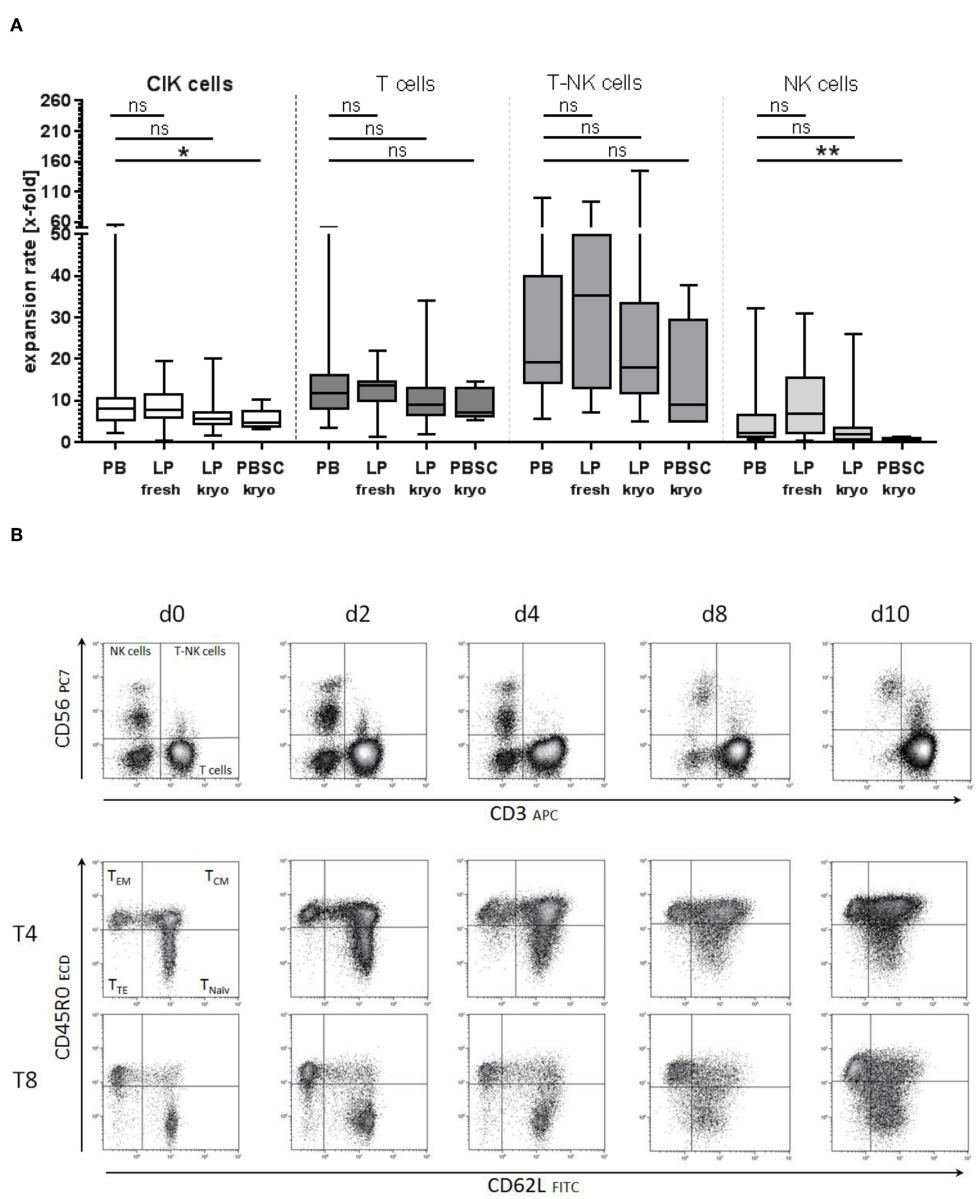
Starting material of CIK cell generation. **(A)** CIK cell generation on the basis of different starting materials showed comparable expansion rates for peripheral blood (PB) and unstimulated leukapheresis products (LP, fresh, and cryopreserved). Significantly lower proliferation was observed when CIK cells were generated from cryopreserved G-CSF stimulated PBSC (*n* = 5). **(B)** No significant differences in the composition of naïve and memory T cells were detected between CIK cells generated from the different starting materials. Exemplarily shown for PB are the subgroups of naïve, central- and effector-memory T cells for cytotoxic and helper T cells on days 0, 2, 4, 8, and 10 generated from PB. T, T cells, EM- effector memory; CM, central memory; TE, terminally differentiated. ^*^p < 0.05, ^**^p < 0.01.

To prove the feasibility of an established clinical- scale expansion protocol, patient doses were generated from PB or LP under class A conditions with subsequent testing of the final product. Initially, for CIK cell generation either ficollized PB or unstimulated LP products were used. Next, cryopreserved LP and PBSC came into the focus of interest. Comparing CIK cell expansion rates, no significant differences were detected between the primary materials PB and LP. However, significantly lower CIK cell expansion rates especially regarding NK cells were identified when granulocyte-colony stimulating factor (G-CSF) stimulated cryopreserved PBSC were the base for CIK cell generation (Figure 4A). Phenotypic analysis showed, that T cells including CD45RA^+^CD62L^+^ naïve, CD45RO^+^CD62L^−^ effector- and CD45RO^+^CD62L^+^ central memory cells did not differ among the different starting materials (Figure 4B, exemplarily shown for PB).

When available, we favored LP [e.g., 1 × 10^9^ white blood cells (WBC)] which we cryopreserved in cryo-bags with adequate cell numbers to be able to generate several clinical doses of 1 × 10^6^ T cells/kG, 5 × 10^6^ T cells/kG, 1 × 10^7^ T cells/kG, and 1 × 10^8^ T cells/kG if applicable. CIK cell doses of 1 × 10^8^ CIK cells per kG body weight required large amounts of cytokines and culture medium, which was not applicable in all patients. Initial number of cells depended on CIK cell expansion rates. A minimum expansion rate of 6-fold was used for calculation (Figure 2B). Recipient body weight in our cohort receiving CIK cells between January 2016 and December 2018 was 22.0 kG (range: 16.6–75.0 kG). With our protocol, a total CIK cells doses of in median 76.7 × 10^6^ T cells/kG (range: 22.7–175.2 × 10^6^ T cells/kG) recipient body weight including 6.9 × 10^6^ T-NK cells/kG (range: 0.5–28.0 × 10^6^ T-NK cells/kG) were provided (n=15).

## Discussion

The application of CIK cells is a promising immunotherapeutic approach to combat relapse, which remains one of the leading causes of treatment failure following allogeneic HSCT for acute leukemia or myelodysplastic syndrome (14, 19, 20). CIK cells are a heterogeneous composition of CD3^+^CD56^−^ T cells, CD3^+^CD56^+^ T-NK cells and a minority fraction of CD3^−^CD56^+^ NK cells (21). CIK cells acquire NK phenotype and activating NK cell receptors such as NKG2D, and NKp30 during *in vitro* expansion. T-NK cells as main effector cells have shown potent killing activity via non-MHC-restricted NK-like cytotoxicity and TCR-dependent pathways with the ability to traffic to tumor sites. Considering the urgent therapeutic need of patients with impending relapse after allogeneic HSCT, we developed a cultural system with which CIK cells can be provided more rapidly (14). By using IL-15, cells with CIK cell phenotype were expanded within 10–12 days. The cytotoxic capacity of IL-15 activated CIK cells, was best between days 10–12 of culture, parallel to the expression of activation antigen CD25. However, compared to the 3 week culture system, numbers of T-NK cells were lower while numbers of CD3^+^CD8^+^TCR α/ß^+^ T-cells were higher, potentially associated with increased risk for GVHD. To increase numbers of T-NK cells and therewith efficacy and to decrease alloreactivity, CD56-enrichment was performed (7). Most interestingly, preclinical *in vitro* and *in vivo* mouse data showed very little, if any alloreactivity, while the bulk CIK cell population showed increased antitumoral activity, with contrasting results obtained by the CD56^+^ subpopulation, consistent to the *in vitro* data showing a drop of cytotoxicity after day 14 despite increasing numbers of T-NK cells. Therefore, T cells also attributed to high value to CIK cell-mediated cytotoxicity and the bulk CIK cell population was considered for clinic use. However, the mechanisms of improved CIK cell function after IL-15 activation is not been well-elucidated yet. Expression of toll-like receptor 4 seemed to be involved in the increased cytotoxic potential of IL-15 activated CIK cells (22). Furthermore, RNA-sequencing showed that the up-regulation of Wnt 4 and PDGFD may contribute to enhanced proliferation of IL-15 activated CIK cells (23). In contrast to our results, Tao et al. demonstrated a down regulation of regulatory T cells, and an increase of T-NK cells and proinflammatory cytokines among IL-15 activated CIK cells compared to IL-2 activated controls (24).

To meet the requirements of cell expansion for clinical use, primary material, culture conditions have to be well-defined to enable manufacturing of CIK cell products in a reproducible, cost-, time- and human resources-saving way. We assessed primary materials and GMP-grade culture media supplements for their ability to support cell proliferation and function in X-Vivo 10 cultures. Furthermore, maintaining cell viability, phenotype and function was assessed after freeze-thawing of generated CIK cell doses.

Altogether, we favored large scale CIK cell generation for individual patients, whom we provided with an initial fresh dose and cryopreserved ready-for-use CIK cell doses. The culture media supplements might show differences in promoting cell proliferation. As a search for alternatives to replace bottlenecks of AB serum given due to the infrequent occurrence of blood-type AB within the population, we tested FFP, PL, and XerumFree^TM^ as cell culture additives. CIK cells cultured with 10% FFP and 5% PL showed comparable phenotype, proliferation, viability and cytotoxic activity compared with CIK cells cultured in the presence of AB serum. With XerumFree^TM^ we tested a serum additive which is generated completely on plant basis. However, XerumFree^TM^-generated CIK cells showed significantly lower proliferation and therefore was not suitable for CIK cell generation. Usta et al. obtained satisfying results while using xeno-free media for multiple cell lineage culture (25). That said, proliferation rates might be more relevant for immune cell generation compared to cell culture. Other groups applied platelet rich or autologous plasma instead of bovine serum in the culture of MSCs and dendritic cells with good results regarding proliferation and cell quality (26, 27). Good results were also obtained using human PL as a serum substitute in cell culture media (28). Furthermore, CIK cell activation and expansion is provided by a well-defined cytokine cocktail. A 10–12 day storage in a closed GMP-compliant cell culture bag leads to nutrient and cytokine exhaustion and accumulation of metabolic products which may impair cell proliferation and function. For reproducible and efficient cell expansion, medium supplemented with AB serum, FFP or PL as well as cytokines were given at defined time points and concentrations and CIK cell concentration was kept at 1 × 10^6^ cells/mL. Our data demonstrated that X-Vivo 10 containing FFP or PL was as affective as AB serum in promoting cell proliferation and viability neither was, there a significant alteration in the CIK cell composition or in their cytotoxic activity against leukemic cell line THP-1 cells. Due to its easy accessibility, we found FFP as an optimal medium supplement supporting cell proliferation and cytotoxicity in a 10–12 batch CIK cell culture, which finally resulted in the establishment of a clinical grade manufacturing procedure enabling us to obtain sufficient numbers of functional cells forming stable ready-for-use patient doses given either fresh after cryopreservation procedure.

Literature lacks information about recovery and cytotoxicity of CIK cells following cryopreservation and thawing. We analyzed the cytotoxic potential of CIK cells following overnight cryopreservation and a storage time of in median 2years with encouraging results. However, CD34^+^ stem cells showed high recovery and viability after cryopreservation allowing safe transplantation with good engraftment results (29, 30), the same applies to donor lymphocyte infusions (DLI) obtained from for example unstimulated LPs. Furthermore, we and others obtained long-term experience in the cryopreservation of mesenchymal stromal cells (31, 32). In a study by Berens et al. the post-thaw recovery of CD34^+^ stem-, B-, T-, and NK cells has been analyzed in 90 apheresis products. Interestingly, with 98.6 ± 15 % B cells showed the best recovery followed by stem cells (93.0 ± 20.7 %) and NK cells (90.4 ± 24 %). CD4^+^ helper and CD8^+^ cytotoxic T cells showed a significantly lower post-thaw recovery than stem cells with 83.1 ± 15.4 and 83.3 ± 13.9 %, respectively (29). Our results for T cell recovery in thawed CIK cells were with 93.3% (range: 70.1–100%) higher than these results, which might be attributed to the cytokine stimulation of CIK cells. This hypothesis might be supported by our former results showing that IL-2 stimulated NK cells were more robust after cryopreservation than unstimulated NK cells (33).

With the aim to reach flexibility and to reduce requests for additional donor cells, we tested different starting materials for CIK cell generation including ficollized PB, fresh LP as well as cryopreserved LP and PBSC. Except cryopreserved PBSC, CIK cells from all starting materials resulted in comparable expansion rates and final product composition. CIK cells generated from cryopreserved PBSC were associated with limited expansion of NK and T-NK cells resulting in their reduced frequency in the final product. This delay in expansion might be attributable to an effect of G-CSF on the cells (34, 35). As the enrichment of CD56^+^ cells within CIK cells seems to correlate with an increased cytotoxic potential, cryopreserved PBSC does not seem to be an appropriate starting material for CIK cell generation (7). Derived from our primary material-data, we preferred LP as starting material for CIK cell generation, as appropriate amounts of 1 × 10^9^ mononuclear cells either fresh or cryopreserved were provided by this material. Exactly portioned cryopreserved mononuclear cells enabled a rapid production of ready-for-use CIK cell doses without 10–12 days when a patient was diagnosed with molecular relapse. Starting from one LP, at least 8–10 CIK cell applications with several clinical doses of 1 × 10^6^ T cells/kG, 5 × 10^6^ T cells/kG, 1 × 10^7^ T cells/kG, and 1 × 10^8^ T cells/kG were generated. However, only the cultivation of large doses of CIK cells especially for adipose patients remained challenging. Pre-defined specification in terms of cell viability (specification >70%), cell expansion of CD3^+^CD56^+^ T-NK cells expressing the activation antigen CD25 (specification >10-fold) and T cells amount (specification <1 × 10^8^/kg), negativity for culturable microbes, mycoplasma and endotoxin were already performed before and during culture procedure so that release as an IMP was not delayed like recently reported by others (36).

To avoid cytokine-related clinical symptoms, generated CIK cells were washed three times before being applied in injection volumes of 100 mL to the individual patient.

CIK cell products generated from donors' peripheral blood or apheresis material, under GMP-conditions, including the usage of FFP as medium additive, given in escalating doses regardless of the donor type showed limited alloreactive potential in 36 patients with relapsing hematological malignancies after allogeneic HSCT (37, 38). One dose of CIK cells was prepared for immediate use, remaining cells were cryopreserved for subsequent infusions resulting in a total of 103 infusions. CIK cell treatment significantly improved immune reconstitution, significantly reduced incidence of relapse and improved survival compared to patients with DLIs. Altogether, manufacturing of CIK cells like described here was feasible even though given in significantly higher doses of T cells compared to conventional DLI treatment. Furthermore, based on this data CIK cell therapy is currently being tested in an open-labeled multicenter phase 2 study to evaluate the safety and efficacy of a sequential administration of donor-derived CIK cells to patients with impending relapse of hematological malignancies after allogeneic HSCT (FFM–CIK-Cell Study 01 study, Eudra-CT: 2013-005446-11).

In this study, we successfully established a GMP-compliant procedure enabling production of therapeutic doses of CIK cells. In summary, we showed that CIK cells retain main features like proliferation, viability, phenotype, and function in FFP supplemented culture conditions, which were preserved after and cryopreservation. Results presented in this study further describe a robust process to expand several therapeutic doses of CIK cells from an unstimulated LP under GMP-compliant conditions. Hence, the standard procedure (ATMP §4b Abs. 3 AMG, license number: PEI.A.11630.01.1) was extended by refinements including the usage of FFP as a medium additive instead of AB serum to overcome supply bottlenecks and the cryopreservation of CIK cells to obtain ready-for-use CIK cells in the desired doses.

## Data Availability

All datasets generated for this study are included in the manuscript and/or the Supplementary Files.

## Ethics Statement

This study was carried out in accordance with the recommendations of the medical ethics committee, with written informed consent from all subjects. All subjects gave written informed consent in accordance with the Declaration of Helsinki. The protocol was approved by the medical ethics committee of the University Hospital Frankfurt (Ref. No. 281/14).

## Author Contributions

MB, SH, and ER conceived and designed the experiments. SE, SB, and AQ performed the experiments. MB, SH, CC, and HB analyzed the data. MB and SH coordinated the research. MB, SH, VK, SE, and L-MP contributed to reagents, materials, analysis tools. MB, SH, and ER wrote the paper. HB, MS, ER, CC, MM, VK, AQ, SE, and L-MP revised the manuscript. PB and TK supervised the research. All authors approved the final version of the manuscript.

### Conflict of Interest Statement

The authors declare that the research was conducted in the absence of any commercial or financial relationships that could be construed as a potential conflict of interest.
