# Supplementary figures and images for: Improving Clinical Manufacturing of IL-15 Activated Cytokine-Induced Killer (CIK) Cells
Source: Front Immunol. 2019 May 31;10:1218. doi: 10.3389/fimmu.2019.01218 (PMC6554420; doi:10.3389/fimmu.2019.01218)

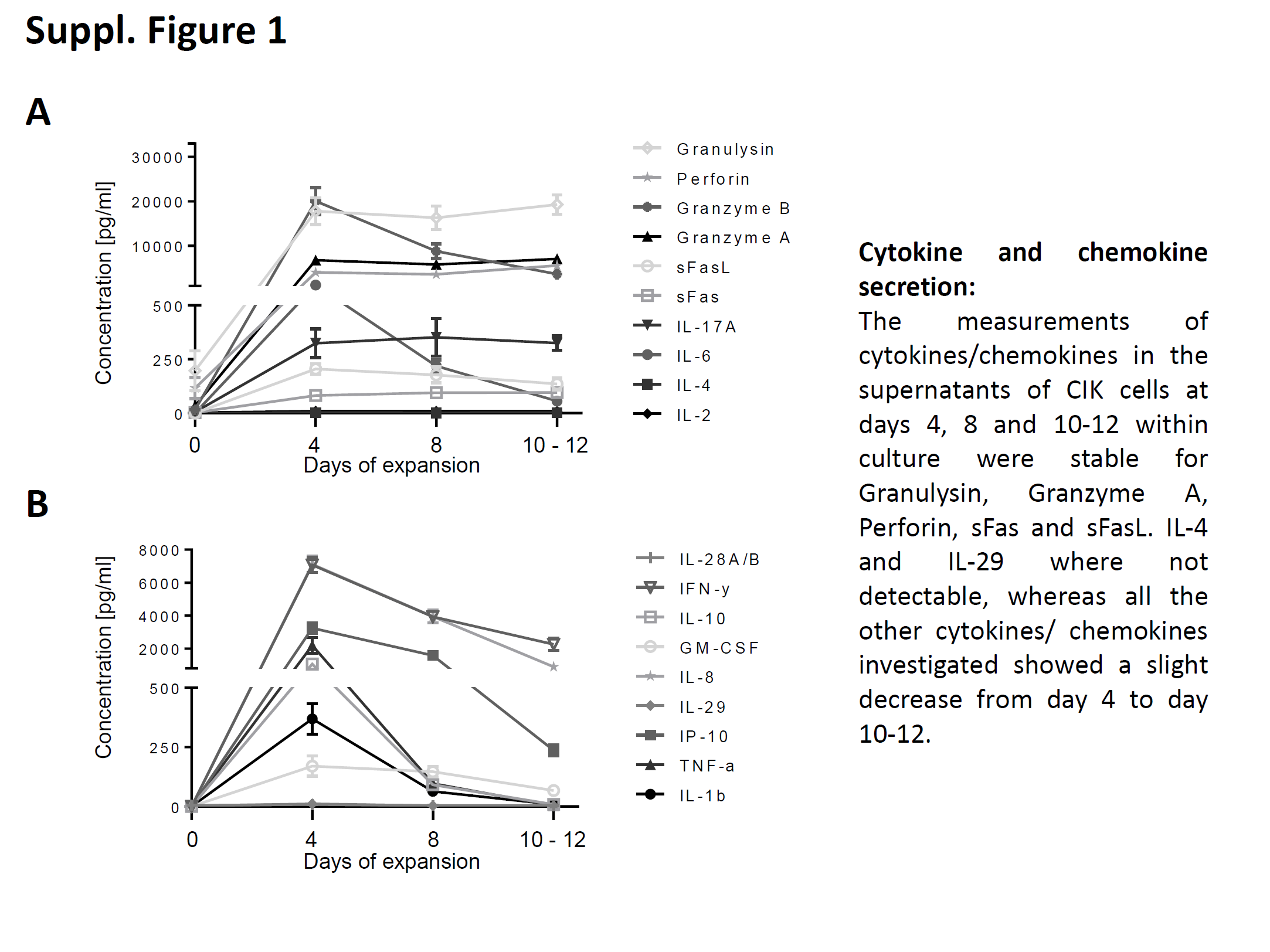

Supplement: Supplementary file 1 [file Image_1.TIF]
